# Riboflavin Deficiency—Implications for General Human Health and Inborn Errors of Metabolism

**DOI:** 10.3390/ijms21113847

**Published:** 2020-05-28

**Authors:** Signe Mosegaard, Graziana Dipace, Peter Bross, Jasper Carlsen, Niels Gregersen, Rikke Katrine Jentoft Olsen

**Affiliations:** 1 Research Unit for Molecular Medicine, Department for Clinical Medicine, Aarhus University and Aarhus University Hospital, 8200 Aarhus N, Denmark; signe.mosegaard@clin.au.dk (S.M.); grazianadipace@outlook.it (G.D.); peter.bross@clin.au.dk (P.B.); jaspercarlsen@hotmail.com (J.C.); nig@clin.au.dk (N.G.); 2 Department of Biosciences, Biotechnology and Biopharmaceutics, University of Bari ‘Aldo Moro’, 70121 Bari, Italy

**Keywords:** riboflavin, riboflavin deficiency, energy metabolism, mitochondria, fatty acid oxidation, acyl-CoA dehydrogenases, inborn errors of metabolism, electron transport chain, folding, MADD

## Abstract

As an essential vitamin, the role of riboflavin in human diet and health is increasingly being highlighted. Insufficient dietary intake of riboflavin is often reported in nutritional surveys and population studies, even in non-developing countries with abundant sources of riboflavin-rich dietary products. A latent subclinical riboflavin deficiency can result in a significant clinical phenotype when combined with inborn genetic disturbances or environmental and physiological factors like infections, exercise, diet, aging and pregnancy. Riboflavin, and more importantly its derivatives, flavin mononucleotide (FMN) and flavin adenine dinucleotide (FAD), play a crucial role in essential cellular processes including mitochondrial energy metabolism, stress responses, vitamin and cofactor biogenesis, where they function as cofactors to ensure the catalytic activity and folding/stability of flavoenzymes. Numerous inborn errors of flavin metabolism and flavoenzyme function have been described, and supplementation with riboflavin has in many cases been shown to be lifesaving or to mitigate symptoms. This review discusses the environmental, physiological and genetic factors that affect cellular riboflavin status. We describe the crucial role of riboflavin for general human health, and the clear benefits of riboflavin treatment in patients with inborn errors of metabolism.

## 1. Introduction

B-vitamins are an important group of vitamins that are vital for crucial cellular functions, such as energy production, and in the formation of other vitamins. One of the key players in these processes is vitamin B2, or riboflavin, and its derivatives, flavin mononucleotide (FMN) and flavin adenine dinucleotide (FAD). The derivatives FMN and FAD play crucial roles as cofactors for enzyme-catalyzed reactions. Riboflavin is a water-soluble essential vitamin present in dietary sources such as meats, milk, fatty fish, nuts, eggs and vegetables, such as spinach and beans, as well as some fruits [1]. Furthermore, riboflavin is frequently added to vitamin enriched products, such as baby foods and cereals, and it has been suggested that some bacteria in the human microbiome are able to produce riboflavin [2].

**Figure 1:**
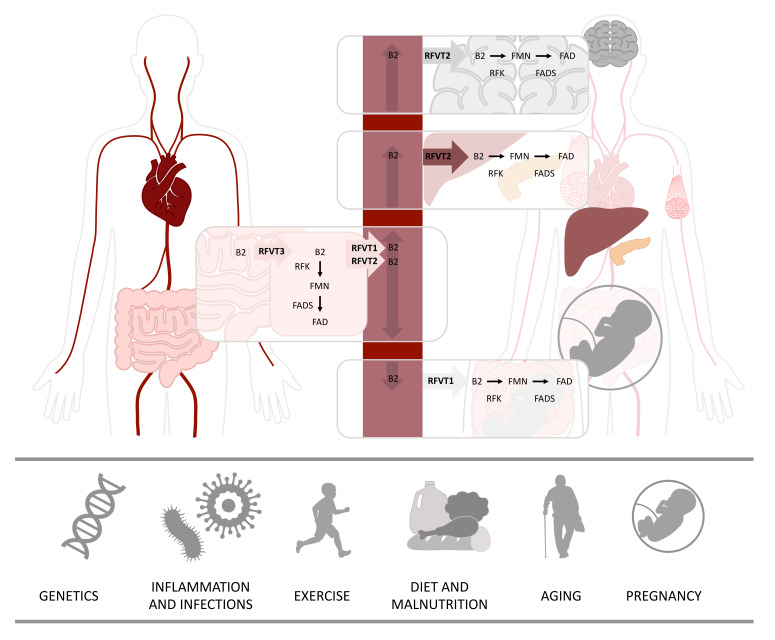
Riboflavin metabolism and its interaction with environmental factors. Riboflavin is absorbed from the gastrointestinal tract predominantly by riboflavin transporter 3 (RFVT3). Inside the gastrointestinal cells, riboflavin can either be further metabolized to flavin mononucleotide (FMN) by riboflavin kinase (RFK) or to flavin adenine dinucleotide (FAD) by FAD synthase (FADS) or transported to the bloodstream by riboflavin transporter 1 (RFVT1) and riboflavin transporter 2 (RFVT2). Riboflavin is distributed via the bloodstream to its destination cells. In addition to being expressed in the gastrointestinal system, RFVT1 is expressed in the placenta, where it carries riboflavin from maternal bloodstream to fetal bloodstream. RFVT2 is expressed all over the body and highly expressed in the brain, endocrine organs, such as pancreas, but also in the liver and muscle tissue. Inside the destination cells, riboflavin is used directly or transformed into either FMN or FAD, which are used as cofactors for several processes. Several factors can affect human riboflavin status, hereunder, genetics, inflammation and infections, exercise, diet and nutrition, aging and pregnancy.

Dietary sources contain free riboflavin, but also contain flavoproteins with bound FMN and FAD that need to be hydrolyzed to riboflavin before this can be absorbed [3]. The absorption and transportation of riboflavin is performed by a group of transporters from the solute carrier family SLC52. The majority of riboflavin is absorbed in the small intestine, but absorption also occurs in the stomach, duodenum, colon and rectum, through an active carrier-mediated transport by riboflavin transporter 3 (RFVT3), illustrated in Figure 1. The main function of RFVT3 is to absorb riboflavin from the diet. Once riboflavin is absorbed, it can be transformed into FMN and FAD, and used by the epithelial cells of the gastrointestinal tract, or it can be transported by riboflavin transporter 1 (RFVT1) or riboflavin transporter 2 (RFVT2) to the bloodstream and distributed to tissues, where it is needed. RFVT2 is distributed all over the body and can be detected in high amounts in the brain, but also in endocrine tissues, liver and muscle. Just like the other two transporters, RFVT1 is expressed in the gastrointestinal tract, but one of its most important roles is to transport maternal riboflavin to the fetus in the placenta. When riboflavin reaches the target cells, it is transformed into its active forms, FMN by riboflavin kinase (RFK), and in turn to FAD by FAD synthase (FADS) [3,4], as illustrated in Figure 1. Abundant amounts of flavins are used by the mitochondria, and both FMN and FAD can be transported inside the mitochondrion by the mitochondrial folate transporter (MFT) [5]. Several studies indicate that the human mitochondrion itself has the ability to produce FAD. In support of this theory, several FAD synthase isoforms have been reported, including an isoform with a mitochondrial targeting sequence [6,7,8,9].

The daily dietary requirements of riboflavin range from 1.0 to 1.3 mg/d in adults, and even higher amounts are advised to young adults, pregnant and lactating women, athletes and elderly people. The excess riboflavin is excreted in the urine as riboflavin or as riboflavin-derived metabolites, 7-hydroxymethylriboflavin and lumiflavin. To date, there are no reported complications associated with riboflavin supplementation, even when supplied in very high doses [3,10]. Several factors can affect human riboflavin status, of which diet has the largest impact in the general population. However, other factors such as pregnancy, exercise, aging, infections—and in rare cases genetic variations—can also affect riboflavin status (Figure 1).

## 2. Riboflavin’s Importance for Cellular Functions

In 1975, Hoppel and Tandler illustrated the importance of riboflavin in the mitochondrial energy metabolism by showing that riboflavin depletion in mice led to a wide impairment of mitochondrial function [11]. Despite its central role in the mitochondrial energy metabolism, other metabolic pathways strongly depend on riboflavin. In fact, the human genome contains 90 genes encoding flavin-dependent proteins, which together form the human flavoproteome [12]. Flavoproteins are involved in various cellular functions, as briefly described below.

A wide number of flavoenzymes are involved in lipid and protein metabolism. Seven mitochondrial acyl-CoA dehydrogenases (ACADs) and three peroxisomal acyl-CoA oxidases (ACOX) are involved in the catabolism of lipids. Flavoenzymes are active also in lipid anabolism, in particular, they participate in fatty acid desaturation and cholesterol metabolism in the endoplasmic reticulum and in ether lipid synthesis in peroxisomes [12,13]. Four ACADs are involved in the mitochondrial catabolism of various amino acids, and choline dehydrogenase (CHDH)*,* dimethylglycine dehydrogenase (DMGDH) and sarcosine dehydrogenase (SARDH) process choline in mitochondria. Flavoenzymes are involved also in the oxidative folding of proteins in the endoplasmic reticulum [12,14].

Riboflavin is crucial for mitochondrial electron transport chain (ETC) function. In fact, complex I and complex II harbor FMN and FAD, respectively, and the mitochondrial glycerol-3-phosphate dehydrogenase (GPDM), the sulfide:quinone oxidoreductase (SQOR), the electron transfer flavoprotein (ETF) and the electron transfer flavoprotein-ubiquinone oxidoreductase (ETF-QO), which shuttle electrons to CoQ10, are all flavoenzymes [12].

In addition, flavoenzymes play a pivotal role in cellular stress responses. Glutathione reductase is a flavoenzyme, which catalyzes the reduction of glutathione disulfide to glutathione, a key cellular antioxidant peptide [12,15]. Flavoenzymes are also involved in cellular apoptosis. The apoptosis inducing factor (AIF) is a mitochondrial flavoenzyme, which during apoptosis is translocated to the nucleus, where it is incorporated into a chromatin-degrading complex, ultimately leading to cell death [16]. The apoptosis-inducing factor-homologous mitochondrion-associated inducer of death (AMID) is a newly characterized cytosolic flavoenzyme involved in caspase-independent apoptosis [12,17]. Finally, flavoenzymes participate in drug metabolism, since both mitochondrial and microsomal cytochromes P450 receive electrons from flavoenzymes [12,18], and in epigenetic regulations, with lysine-specific demethylase 1 (LSD1) utilizing FAD as cofactor [12,19].

### The Role of Riboflavin in Homocysteine, Folic Acid and the Metabolism of Other Vitamins

Homocysteine is an amino acid derived from methionine, and high levels of homocysteine are associated with a risk of developing cardiovascular diseases [20]. Metabolizing homocysteine can be performed in two ways, either transsulfuration, depending on vitamin B6 (pyridoxine), or remethylation, depending on vitamin B9 (folic acid), vitamin B12 (cyanocobalamin) and riboflavin [21]. In the metabolic regulation of homocysteine and the metabolism of folate, the methylenetetrahydrofolate reductase (MFTHR) is a key enzyme. MFTHR uses FAD as a cofactor to metabolize vitamin B9 in the homocysteine methylation. Studies have shown that 5–30% of the general population is homozygous for a common genetic variant (c.677C>T) that is associated with increased levels of homocysteine [22]. Further investigations of this common variant have shown that a daily dose of riboflavin (1.6mg/d) for a 12-week period significantly decreases the amount of homocysteine in plasma in individuals homozygous for the c.677C>T variant. The decrease of homocysteine level could lower the risk of developing cardiovascular disease in the c.677C>T homozygous individuals [23]. Similar to MFTHR, pyridoxine 5′-phosphate oxidase (PNPO), converting vitamin B6 to the biologically active form, pyridoxal phosphate, depends on a riboflavin derivative, in this case FMN [24], and the methionine synthase reductase (MTRR), involved in the regeneration of vitamin B12, uses both FMN and FAD [12]. Even in the biosynthesis of D vitamin, flavoenzymes have important roles [13].

## 3. Riboflavin Deficiency and its Significance in the General Population 

### 3.1. Riboflavin and Diet

Riboflavin deficiency is the most common vitamin deficiency in developing countries with diets lacking riboflavin-rich products, such as milk and meat [25]. Clinical symptoms of riboflavin deficiency appear only after several months of insufficient riboflavin intake, and vary from milder symptoms as sore throat, loss of hair, and skin inflammation, to severe symptoms as swollen tongue, anemia and impaired nerve functions. Although these symptoms are rarely seen in non-developing countries and well-nourished societies, dietary insufficiency and subclinical riboflavin deficiency is detected in remarkably large groups in the population [26,27,28,29]. Several population studies of vitamin status report on riboflavin insufficiency in children and young adults, especially in young women [30]. A national survey performed in the United Kingdom, investigating the biochemical riboflavin status in 2127 schoolchildren, revealed a poor riboflavin status that increased with age. In boys, from 59% insufficient riboflavin intake in 4–6-year-olds, to 78% in 7–10-year-olds, but the largest group with riboflavin insufficiency were the 15–18-year-old girls. The survey revealed that 95% of the 15–18-year-old girls had an insufficient intake of riboflavin [30] and an increasing risk of developing riboflavin deficiency. The increase of riboflavin insufficiency in both boys and girls is comparable to a declined consumption of milk, from 25% of the daily riboflavin intake in the 4–6-year-olds to only 10% of the daily riboflavin intake in the 15–18-year-olds. The implications for this riboflavin insufficiency, especially in young girls, are not fully known, but it has been shown that subclinical riboflavin deficiency could influence iron handling and that a daily supplement with riboflavin (2 or 4 mg) for 8 weeks significantly improves the hematologic status, with an increase in circulating red blood cells and hemoglobin concentrations in young women, even without an additional iron supplementation [28].

Most of the reported population studies performed on riboflavin status are older studies and the recent year’s changes in lifestyle, especially in well-nourished societies, with diets based on less dairy and meat products in combination with more exercise (see below), could potentially increase the risk of riboflavin deficiency. In this context, studies on the dietary intake of riboflavin in well-nourished countries amongst people that follow a vegan diet without meat, dairy products and eggs, have shown that up to 48% have lower than the recommended daily intake of riboflavin, and thereby an increasing risk for developing riboflavin deficiency [31,32,33].

### 3.2. Riboflavin and Aging

In addition, in the elderly population a general riboflavin insufficiency has been described. Studies in the United Kingdom and the United States indicate that 10–41% of the elderly population have an insufficient riboflavin intake [34,35] and are at risk of developing riboflavin deficiency, based on dietary reports [36]. The deficiency observed in the elderly population in these studies can partly be explained by a decreased intake of milk and other dairy products. However, the most plausible explanation is that the elderly population displays a reduced efficiency in the absorption of riboflavin that increases with aging [3,37]. In addition, riboflavin deficiency and deficiency of other B vitamins in the elderly have been linked to depression and changes in cognitive function, and it has been shown that riboflavin supplementation in elderly people could work as a neuroprotective agent and prevent disorders such as dementia, Parkinson’s and Alzheimer’s disease [38,39,40,41,42].

### 3.3. Riboflavin and Exercise

Athletes and people with high physical activity could be at risk of developing riboflavin deficiency. Studies in healthy men with a moderate activity level and biochemical signs of riboflavin deficiency have shown that even short periods with increased physical activity deteriorate riboflavin levels further. The deterioration in riboflavin is caused by the metabolic stress that occurs during periods of increased physical activity [43,44].

### 3.4. Riboflavin Pre- and Postpartum

One of the groups at high risk of developing riboflavin deficiency is women during pregnancy, where the needs of riboflavin rise with fetal development and continue postpartum. Riboflavin deficiency may not only affect the mother but also her child.

In 1943, the first connections between women’s diet and their pregnancy were reported by Burke et al. [45]. They observed that maternal nutritional status affected the infant’s condition at birth. Today, the connection between maternal nutritional status and normal fetus development and growth is well described, and it is known that many vitamins are of huge importance during pregnancy, including riboflavin. Riboflavin is essential for normal fetal development, and animal studies have shown that severe riboflavin deficiency in pregnant mice and chicken leads to abnormal fetal development and termination of pregnancy [46,47]. In humans, most studies documenting riboflavin deficiency have been performed in societies with low riboflavin intake. The studies have shown that the risk of riboflavin deficiency in pregnant women is especially high during the third trimester, approaching parturition and during lactation. During pregnancy, the metabolic needs increase, with an average of 230 kcal pr. day, which for most women is more than 10% of their total daily kcal intake. In the first two trimesters the anabolism is dominating, there is an increased insulin sensitivity, and the maternal fat deposits increase [48]. At the beginning of the third trimester, hormones from the placenta cause an increasing insulin resistance and catabolism in the mother, that enable nutrients for fetal growth, and an increasing need for energy rich nutrients, such as fatty acids and vitamins, including riboflavin, to ensure mitochondrial energy metabolism [49,50]. The increased need of nutrients and supporting vitamins, such as riboflavin, continues during parturition and postpartum. During parturition, riboflavin has an important antioxidant function, based on FAD being an essential cofactor for glutathione. Glutathione is crucial for counteracting the peroxidation reactions triggered by the rapid change from a hypoxic to a hyperoxic environment during birth [51]. Riboflavin is important also postpartum. Studies have shown that light therapy, that is frequently used for treating hyperbilirubinemia in infants shortly after birth, can cause riboflavin deficiency and lowers riboflavin levels to 50% within hours [52]. Moreover, maternal nutrient and riboflavin status is of great importance during breastfeeding. For infants that are exclusively breastfed, maternal milk is the only source of riboflavin. Riboflavin is mostly present as FAD in human milk and maternal riboflavin deficiency is rapidly reflected in low flavin concentration in the milk [53]. The postpartum breastfeeding of the infant is of great importance to ensure nutrient needs, but also to develop the infant’s immune response.

### 3.5. Riboflavin and the Immune Response 

Riboflavin, FMN and FAD play a key role in immune functions and responses as described recently by Suwannasom et al. [54]. FMN and FAD are important cofactors for the human energy metabolism that is closely connected to the cellular immune responses. The immune response requires tremendous amounts of ATP since a large number of cells need to either be differentiated, proliferated or activated in order to perform their function. Both FMN and FAD are exceedingly involved in the production of ATP, as cofactors for crucial flavoenzymes in the oxidation of fatty acids and branched-chain amino acids, in the Krebs cycle and in the ETC. Recently, riboflavin was shown to have an important role in macrophage function, and that riboflavin deficiency causes disruption in the activation of macrophages that ultimately leads to a decreasing recognition of pathogens and a failed activation of immune responses [55]. Additionally, the key producers of ROS in the immune response, the nicotinamide adenine dinucleotide phosphate (NADPH) oxidases (NOX), require binding of FAD. The production of ROS is crucial for destroying pathogen DNA, RNA and proteins, and ROS have an important role as cellular signaling molecules of immune cell function [56,57].

Several studies have investigated the functions of riboflavin in the immune responses, and riboflavin has been reported to have anti-inflammatory effects by lowering several proinflammatory cytokines, namely TNF-α, IL-1ß and IL-6 [58,59]. Moreover, riboflavin can reduce mortality in mice with septic shock, and it has been suggested that riboflavin treatment in septic shock could be potentially useful also in humans [58]. Using riboflavin as a treatment in disease is not new, and in the field of inborn errors of metabolism (IEM), riboflavin treatment has been used extensively and shown extraordinary results in some groups of patients.

## 4. Inborn Errors of Metabolism—Riboflavin as a Potent Treatment

Riboflavin supplementation is a well-established therapy in some IEM, such as multiple acyl-CoA dehydrogenation deficiency (MADD) and riboflavin transporter deficiencies, leading to a significant clinical improvement or stabilization in the majority of patients. Riboflavin supplementation has shown promising results in other IEM, but only single case reports or small-scale studies are reported in the literature. Therefore, further investigations and large-scale clinical trials are needed to confirm riboflavin therapy efficacy in those patients. An overview of IEM benefiting from riboflavin supplementation can be found in Table 1.

Riboflavin responsiveness can be explained by three major molecular mechanisms, although overlapping mechanisms may exist in individual IEM.

**Figure 2:**
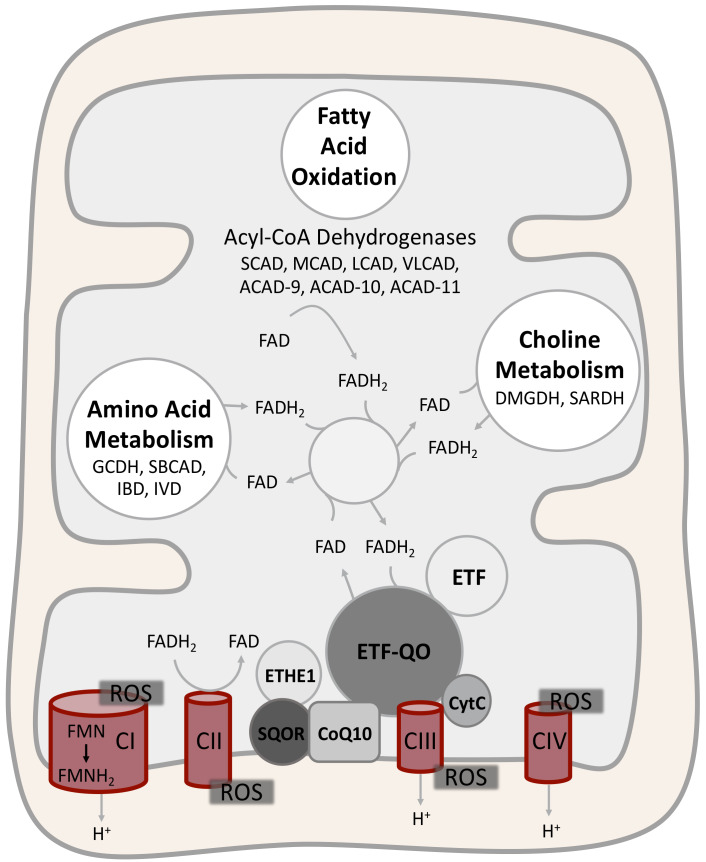
Disease-related flavoenzymes in mitochondrial energy metabolism. Several flavoenzymes are involved in the mitochondrial energy metabolism including fatty acid oxidation, amino acid metabolism and choline metabolism. Acyl-CoA dehydrogenases are involved in fatty acid oxidation and amino acid metabolism, while dimethylglycine dehydrogenase (DMGDH) and sarcosine dehydrogenase (SARDH) participate in choline metabolism. All these dehydrogenases harbor FAD as a redox cofactor, which is reduced to FADH_2_ during the dehydrogenation reactions. The dehydrogenases donate the electrons obtained from their substrates to the electron transfer flavoprotein (ETF), and finally to the electron transport chain (ETC) through the ETF-ubiquinone oxidoreductase (ETF-QO), which reduces coenzyme Q10 (CoQ10). The ethylmalonic encephalopathy protein 1 (ETHE1) is connected to the electron transport chain via the sulfide:quinone oxidoreductase (SQOR) and donates electrons to CoQ10. In the assembly and function of complex I and complex II, FMN and FAD, respectively, have important functions. Additionally, both FMN and FAD are crucial for folding and stability of all these flavoenzymes, that, in some cases, explains the benefits of riboflavin supplementation in patients with genetic variants causing primary or secondary dysfunction of flavoenzymes. Riboflavin can also compensate secondary flavin homeostasis derangements. Abbreviations: ACAD9, acyl-CoA dehydrogenase family member 9; ACAD10, acyl-CoA dehydrogenase family member 10; ACAD11, acyl-CoA dehydrogenase family member 11; CI, complex I; CII, complex II; CIII, complex III; CIV, complex IV; CoQ10, coenzyme Q10; CytC, cytochrome C; DMGDH, dimethylglycine dehydrogenase; ETF, electron transfer flavoprotein; ETF-QO, ETF-ubiquinone oxidoreductase; ETHE1, ethylmalonic encephalopathy protein 1; GCDH, glutaryl-CoA dehydrogenase; IBD, isobutyryl-CoA dehydrogenase; IVD, isovaleryl-CoA dehydrogenase; LCAD, long-chain acyl-CoA dehydrogenase; MCAD, medium-chain acyl-CoA dehydrogenase; SARDH, sarcosine dehydrogenase; SBCAD, short-branched chain acyl-CoA dehydrogenase; SCAD, short-chain acyl-CoA dehydrogenase; SQOR, sulfide:quinone oxidoreductase; VLCAD, very long-chain acyl-CoA dehydrogenase.

- It has been shown that riboflavin-derived cofactors are able to bind apoproteins and chaperone their folding (see below). Therefore, increased cellular levels of FMN and FAD could improve folding and stability of mutant flavoenzymes and decrease their proteolytic degradation. This process is likely to happen in inborn genetic errors of flavoenzymes such as ETF, ETF-QO, and certain ACADs, as well as in inborn genetic errors of ETC function and assembly (Figure 2).
- Riboflavin-derived cofactors may counteract secondary impairment of flavoenzymes induced by an IEM. This seems to be the mechanism of riboflavin-responsiveness in genetic disorders of ethylmalonic encephalopathy protein 1 (ETHE1), succinate dehydrogenase assembly factor I (SDHAF1) and mitochondrial tRNA^Leu(UUR)^ (MT-TL1).
- Genetic deficiencies in RFVTs, MFT and FADS invariably affect cellular flavin supply and distribution (Figure 1). In this case, riboflavin supplementation could act directly by restoring cellular flavin levels. Secondary derangements of flavin homeostasis, induced by the variant flavoenzymes themselves (FADS and ETF-QO), and/or by external factors as discussed above, could be equally compensated through this mechanism.

Before we summarize evidence of riboflavin therapy in individual IEM, we will discuss the molecular mechanisms by which flavin cofactors maintain the functional stability of flavoproteins and their genetic variants.

### 4.1. The Role of Flavin Cofactors for Folding and Stability of Flavoenzymes and Their Genetic Variants 

The availability of flavin cofactors plays an important role for both folding and cellular stability of flavoenzymes with the two processes being widely intertwined. During folding, the protein forms all the non-covalent interactions between atoms of the polypeptide chain and the flavin cofactor that are present in the native conformation. The sum of these interactions determines the biophysical stability of the protein. High biophysical stability protects proteins from degradation, as intracellular proteases have a strong preference for unfolded or partially unfolded protein molecules [60]. Therefore, the contribution of the interactions between the atoms of the protein and the atoms of cofactors, like FAD and FMN, have a great influence on the intracellular stability and turnover of flavoenzymes.

Atomic interactions of the polypeptide chain with prosthetic groups often also play a crucial role for the folding propensity. According to the ‘nucleation condensation’ theory of protein folding, one important rate-limiting step for the acquisition of the native structure is the formation of the ‘folding nucleus’, a subset of native interactions [61]. Once formed, the folding nucleus speeds up the formation of the remaining interactions. In the 1990s, experimental evidence for the crucial role of FAD for folding, assembly and stability of the ACADs was provided by the Tanaka laboratory. Firstly, import of various ACADs into rat mitochondria resulted in increased degradation of the ACADs when FAD-depleted mitochondria were used, compared to mitochondria with normal FAD levels [62]. Secondly, follow-up investigations with one of the ACADs, medium-chain acyl-CoA dehydrogenase (MCAD), showed that its folding, after import into the mitochondrial matrix, occurs via sequential interactions with the mitochondrial matrix HSP70 chaperone orthologue and HSP60. The authors demonstrated that FAD was required for the formation of the core structure, a kind of folding nucleus, before MCAD monomers were released from the HSP60 complex and then assembled into native tetramers [63]. This clearly showed that the interactions of the folding polypeptide chain with the flavin cofactor is crucial for folding and assembly of these proteins. Using another mitochondrial ACAD, DMGDH, the Barile group showed evidence of a direct interaction between DMGDH and FADS, with cofactor transfer occurring in a sort of “FAD-chaperoning” action catalyzed by FADS. They also showed evidence of an interaction with the HSP60/HSP10 chaperone complex and a possible control exerted by the redox status of FAD, although the exact mechanisms need further investigations [64].

Except for 6 out of the 90 human flavoenzymes, the flavin cofactors FAD or FMN are non-covalently bound to the protein [12]. The FAD dissociation constant for the human ETF, a heterodimer with one non-covalently bound FAD, has been measured to be 0.04 µM [65], i.e., the great majority of ETF molecules have FAD bound under physiological conditions. The melting temperature, a parameter of conformational stability, of some purified ACADs, was shown to be significantly increased in the presence of FAD, suggesting that the availability of another FAD molecule, quickly replacing the dissociated one, protects against denaturation [66]. The presence of the substrates of these enzymes had an additional stabilizing effect. The residual activity of these enzymes at a fever simulating temperature (40 °C) was also stabilized by presence of FAD and additionally by presence of both FAD and the preferred substrate.

The majority of disease-associated point variants in flavoenzymes impair folding and conformational stability [67]. In the light of its role for folding and stability, the levels of the flavin cofactor become even more crucial as this can modulate the residual function of the affected enzymes both by spurring correct folding and by stabilizing the native structure. Two in vitro studies on genetic variants of ETF support this notion. ETF with a disease-causing variant in the beta subunit (ETFβ-p.Asp128Asn) lost the bound FAD more readily at 39 °C, a temperature modeling fever, and the presence of saturating levels of FAD alleviated this [65]. Similar ‘fever’ treatment of two ETF variant proteins differing in a polymorphic site (Ile or Thr at position 171) in the alpha subunit showed that the less frequent variant lost the FAD more readily. Furthermore, the presence of the bacterial HSP60 chaperonin GroEL protected ETF activity under these conditions [68].

In a study from our research lab [69], we used a human HEK-293 cell expression system to investigate the influence of riboflavin and temperature on the steady-state level and the activity of variant ETF-QO proteins, identified in patients with riboflavin-responsive or non-responsive MADD disease. All studied variants were of the missense type and they showed temperature-dependent impairment of ETF-QO protein stability and function to varying degree. However, only those variants (p.Gly429Arg, p.Pro456Leu, p.Pro483Leu) associated with a riboflavin-responsive clinical phenotype could be rescued to wild-type ETF-QO protein and activity levels when expressed at therapeutic concentrations of riboflavin. One of the variants (p.Pro456Leu) showed indications of prolonged association with HSP60, when imported into rat liver mitochondria. The results from these experimental analyses, and presumed structural effects of ETF-QO missense variants [70], are consistent with the idea that the riboflavin response results from the ability of FAD to act as a chemical chaperone that can promote folding and stability of certain variant ETF-QO proteins. This is in accordance with recent biophysical and structural studies of the purified Rhodobacter p.Pro389Leu ETF-QO variant protein, a model for the disease-associated human p.Pro456Leu ETF-QO variant. This variant, as compared to wild-type protein, showed lower stability of FAD binding and increased susceptibility to thermal unfolding, FAD loss and proteolytic degradation [71]. Interestingly, the p.Pro456Leu variant, along with other riboflavin-responsive variants, is located in the ubiquinone-binding domain of ETF-QO. Yet, other riboflavin-responsive variants are located in the FAD binding domain. In the HEK-293 expression study described above, we showed that the effects of amino acid variants in the FAD and ubiquinone binding domain are similar; probably due to cooperative folding of the two domains in the early stages of the folding process [69].

Similar to ETF and ETF-QO variants, FADS variants have been associated with various degree of clinical responsiveness to riboflavin. In particular, patients harboring missense variants in the C-terminal domain of FADS have shown a dramatic response [9]. In vitro overexpression with a molar excess of FAD in *E. coli* resulted in a fully FAD-bound p.Ser495del FADS protein and rescued its proteolytic degradation, supporting a chaperone-like action of FAD [9].

Taken together, these studies illustrate that high levels of FAD and FMN, spurred by intake of the precursor riboflavin, promote folding and stability of flavoproteins, especially under stress conditions, like fever. Riboflavin supplementation does even allow certain mutant flavoenzymes to reach a folding efficiency and stability that attenuates the enzyme deficiency.

### 4.2. Inborn Errors of Flavoenzymes

#### 4.2.1. Multiple Acyl-CoA Dehydrogenation Deficiency

The first riboflavin-responsive IEM was described in 1982 as a mild form of MADD (OMIM #231680). MADD is a rare recessive disorder of fatty acid, amino acid, and choline metabolism [72]. Mitochondrial electron transfer from fatty acid, amino acid and choline metabolism depends on a coordinated function of multiple flavoenzymes, many of which are ACADs. The first step of mitochondrial fatty acid oxidation is catalyzed by seven ACADs with different substrate specificity, namely short- (SCAD), medium- (MCAD), long- (LCAD), very long- (VLCAD) chain acyl-CoA dehydrogenase and the more recently characterized acyl-CoA dehydrogenase family members 9, 10 and 11 (ACAD9, ACAD10, ACAD11) [73]. Four ACADs are involved in amino acid metabolism; isovaleryl-CoA dehydrogenase (IVD), isobutyryl-CoA dehydrogenase (IBD), short/branched-chain acyl-CoA dehydrogenase (SBCAD) and glutaryl-CoA dehydrogenase (GCDH) [73]. All the ACADs donate the electrons obtained from their substrates to ETF. ETF transfers the electrons to ETF-QO which in turn reduces CoQ10 in the ETC. Two flavoenzymes involved in choline metabolism (DMGDH and SARDH), also deliver electrons to the ETC through the ETF/ETF-QO enzymes (Figure 2). Inborn errors in ETF subunits (*ETFA*, *ETFB*) or in ETF-QO (*ETFDH*) are the most common causes of MADD. The clinical presentation of MADD is heterogeneous with phenotype severity being related to the effect of variants on protein function. In severe-MADD patients (S-MADD), the disease onset is neonatal, there is a multisystem involvement, and the patients usually die within the first year of life. S-MADD patients are mainly homozygous or compound heterozygous for loss-of-function (LOF) variants. In mild-MADD patients (M-MADD), the disease can have a later onset and it is often triggered by stress conditions. M-MADD patients usually carry missense variants affecting protein folding and stability and have milder clinical symptoms, such as muscle weakness, hypoglycemia, encephalopathy and, more rarely, cardiomyopathies [74]. Interestingly, decreased levels and/or activities of ACADs and ETC complexes, sometimes combined with low levels of flavin cofactors, have been described in patients with ETF or ETF-QO deficiencies, suggesting that a secondary flavin homeostasis derangement occurs [75,76,77,78,79]. However, how ETF/ETF-QO impairment triggers flavin derangement has yet to be established. A possible explanation could be a negative feedback control on riboflavin transport and metabolism, as hypothesized for patients with inborn genetic errors in FADS, and observed in *C. elegans* experiments (see below). Moreover, CoQ10 depletion and massive ROS production have been measured in MADD patients, suggesting that an even wider metabolic reprogramming occurs [80,81]. Riboflavin supplementation has shown excellent results in M-MADD patients, while it has no or very limited effect in S-MADD patients. Most riboflavin responsive patients harbor *ETFDH* missense variants, but some responsive patients with *ETFA* and *ETFB* variants have been reported [75,82]. Riboflavin supplementation could compensate for disturbances of flavin homeostasis induced by endogenous or external sources, and thereby stabilize mutant ETF/ETF-QO folding, as discussed above [69,77].

#### 4.2.2. Short-Chain Acyl-CoA Dehydrogenase Deficiency

Riboflavin responsiveness in SCAD deficiency (SCADD; OMIM #201470) has been investigated in a few studies. In 1995, Kmoch et al. [83] reported on a 5-years-old patient, who experienced seizures, hypotony, lethargy and cyclic vomiting during stress conditions. Genetic analyses revealed homozygosity for the polymorphic c.625G>A variant, which confers susceptibility to SCADD [84]. When riboflavin supplementation (25 mg/kg/d) was started, the condition of the child improved dramatically and after 2 years of riboflavin treatment, biochemical parameters were normalized. Other single case reports on the beneficial effect of riboflavin supplementation in SCADD patients are available, but genetic characterization of the patients was not reported [85,86]. A wider investigation was performed in 2010 by Van Maldegem et al. [87]. The study included 16 symptomatic SCADD children divided into three groups according to their genotype. The first group included patients homozygous or compound heterozygous for rare missense variants, the second group included compound heterozygous patients with one rare missense variant on one allele and the c.625G>A susceptibility variant on the other allele, the third group included patients homozygous for c.625G>A susceptibility variant. Riboflavin supplementation 10 mg/kg/d (up to 150 mg/d) was administered for 35 days. A mild biochemical improvement was observed in five patients belonging to the second group, and four of them reported a clinical improvement as well. However, the riboflavin effect was questionable because the improved clinical condition persisted after discontinuation of treatment in all four patients. This result is in contrast with the clear clinical amelioration observed in Kmoch et al. patient, suggesting that further studies are needed to clarify the effectiveness of riboflavin supplementation in SCADD patients.

#### 4.2.3. Medium-Chain Acyl-CoA Dehydrogenase Deficiency

Some studies testing riboflavin therapy in patients with MCAD deficiency (MCADD; #201450) have been published. A first study was performed by Duran et al. in 1992 [88]. Five patients were given riboflavin supplementation 50–150 mg/d for 3 weeks and MCAD enzyme activity was measured in lymphocytes before and after riboflavin treatment. Upon treatment with riboflavin, residual MCAD activity was raised from 5.2% to 19% in four patients, and from 11.8% to the heterozygous level in the fifth patient. However, neither the clinical outcome nor genetic characterization of the patients were reported. A second study was published in 2014 by Derks et al. The research group examined lipid and protein oxidative damage and antioxidant defense status in blood samples from 27 MCADD patients harboring homozygous or compound heterozygous missense variants [89]. The patients received either no supplementation (14/27), carnitine supplementation (7/27) or carnitine and riboflavin supplementation (6/27). Riboflavin dosage was 50-150 mg/d. Compared with healthy controls, non-supplemented patients and carnitine-supplemented patients displayed increased protein oxidative damage and altered antioxidant defense, while carnitine and riboflavin-supplemented patients did not display any alteration, suggesting an antioxidant activity of riboflavin. The authors hypothesized that FAD chaperone activity could stabilize mutant MCAD and thereby reduce the amount of acyl-CoA derivatives, which have been shown to cause mitochondrial impairment and ROS production. The clinical outcomes were not evaluated. Therefore, further clinical trials are needed to evaluate whether riboflavin could have a beneficial effect in MCADD patients.

#### 4.2.4. Acyl-CoA Dehydrogenase-9 Deficiency

Riboflavin therapy has been successfully tested in mitochondrial complex I deficiency nuclear type 20 (MC1DN20; OMIM #611126, also known as ACAD9 deficiency). The clinical outcome of ACAD9 deficiency is, in most cases, hypertrophic cardiomyopathy, lactic acidosis and muscle weakness [90,91]. Recently, Repp et al. published an overview of riboflavin therapy efficacy in ACAD9 deficiency [91]. Out of 70 patients analyzed, 31 were following a riboflavin treatment and 20/31 reported a clinical improvement. Details on individual riboflavin dosage are not reported, but the authors advise 20 mg/kg/d, 200 mg/d maximum. Moreover, a significant increase in complex I activity was observed in fibroblasts derived from 9/15 patients, when the cells were supplemented with riboflavin. Interestingly, all patients responding to riboflavin treatment harbored missense variants, suggesting that a FAD chaperoning effect could be the molecular rationale of riboflavin responsiveness, maybe combined with a stabilizing effect of flavins on complex I and complex IV assembly and supercomplexes formation [90,91]. 

### 4.3. Inborn Errors Causing Secondary Impairment of Flavoenzymes

#### 4.3.1. Mitochondrial Complex II Deficiency

Riboflavin treatment has been tested also in patients with mitochondrial complex II (or succinate dehydrogenase, SDH) deficiency (OMIM #252011) [92], but only a small number of patients benefited from the treatment, as reviewed in Jain-Ghai et al. [93]. The three patients benefiting from riboflavin treatment [92] carried a homozygous missense variant in *SDHAF1*, encoding SDHAF1, which contributes to iron-sulfur cluster incorporation into the B subunit of complex II (SDHB) [94]. The mechanism underlying riboflavin responsiveness in these patients was later explained by Maio et al [95]. Experiments in patients’ cells, demonstrated that impaired SDHB biogenesis resulted in lower SDH subunit A (SDHA) flavinylation and protein level. When the cells were supplemented with riboflavin, SDHA protein level and flavinylation increased, together with complex II activity [95]. These experiments demonstrate that riboflavin supplementation is beneficial and should be attempted in patients with complex II deficiency and *SDHAF1* variants.

#### 4.3.2. Ethylmalonic Encephalopathy

Pathogenic variants in *ETHE1*, encoding the mitochondrial persulfide dioxygenase ETHE1 [96], cause ethylmalonic encephalopathy (EE; OMIM #602473), an extremely rare disorder of mitochondrial sulfur metabolism [97]. The disease manifests neonatally, usually with fatal outcome within the first years of life. Patients present with a complex and diverse range of severe symptoms of encephalopathy, vasculopathy and enteropathy, and a characteristic biochemical profile with tissue specific complex IV deficiency, lactic acidemia and elevated excretion of ethylmalonic acid, thiosulfate, C_4_- and C_5_-acylglycines and C_4_- and C_5_-acylcarnitines [97,98,99]. The etiology of these biochemical features was more than a decade later revealed to be due to secondary inhibition of complex IV and SCAD by the toxic accumulation of the gasotransmitter hydrogen sulfide [97]. A resemblance of some EE biochemical features with ACADs and complex IV deficiency may have encouraged treatment with riboflavin. While some reports find no effect of riboflavin [98,100,101,102,103,104,105,106,107], a number of reports suggest some level of positive effect [99,108,109,110,111,112]. However, the therapeutic effect of riboflavin is difficult to interpret since most studies have used riboflavin in combination with other vitamins or cofactors. A beneficial effect of riboflavin may be well founded. Prior to the reaction catalyzed by ETHE1, sulfide is oxidized to persulfide in a reaction catalyzed by the flavoenzyme SQOR, where it donates electrons directly to CoQ10 in the ETC [113,114]. Proper function of complex IV, and the ETC in general, is therefore instrumental in the immediate first step of sulfide clearance. The accumulation of sulfide both destabilizes and inhibits the activity of complex IV [115], meanwhile, riboflavin supplementation may aid in counteracting this destabilization by providing flavin cofactors needed for complex IV assembly factor stability [116,117]. Moreover, riboflavin supplementation may also stabilize SCAD, counteracting the destabilization induced by sulfide. Today no curative treatment of EE has been described, but multiple complementary life extending treatments for managing EE are available [118,119,120,121,122]. Treatment with riboflavin can be used in combination with these and may be beneficial.

#### 4.3.3. MT-TL1 Deficiency

*MT-TL1*, encodes the mitochondrial tRNA^Leu(UUR)^. Various clinical outcomes of *MT-TL1* variants have been described, ranging from pure myopathy to MELAS syndrome (OMIM #540000), and some patients responding to riboflavin have been reported [123,124,125,126]. An interesting study, by Garrido-Maraver et al. [127], demonstrated that riboflavin or CoQ10 supplementation rescued ETC function in fibroblasts obtained from a MELAS patient with a heteroplasmic variant in *MT-TL1*. Supplementation with either riboflavin or CoQ10 resulted in a significant increase in ATP levels and respiratory chain function, and a considerable decrease in superoxide production and mitochondrial degradation. Beneficial effects of riboflavin and CoQ10 supplementation were observed in MELAS cybrids with the same variant. The beneficial effects of riboflavin or CoQ10 supplementation can be most likely attributed to their antioxidant activity, which prevents ROS production and mitophagy. The results obtained by Garrido-Maraver et al. are very promising, but clinical trials are needed to confirm their in vitro studies.

### 4.4. Disorders of Flavin Transportation and Biosynthesis

#### 4.4.1. Riboflavin Transporter Deficiencies

Riboflavin transporter deficiency, which includes Brown-Vialetto-Van Laere syndrome (BVVLS1; OMIM #211530 and BVVLS2; OMIM #614707) and Fazio-Londe disease (OMIM #211500), is caused by variants in *SLC52A2* and/or *SLC52A3,* which encode the human riboflavin transporters RFVT2 and RFVT3, respectively. Even if some clinical differences in patients with *SLC52A2* or *SLC52A3* variants have been reported, the most frequently observed phenotype is a progressive peripheral and cranial neuropathy with vision loss, deafness, sensory ataxia, muscle weakness and respiratory compromise [128]. Some patients show a MADD-like acyl-carnitine profile, reflecting that ETF/ETF-QO and the associated ACADs all depend on riboflavin for proper function [129]. Riboflavin supplementation (10–80 mg/kg/d) is widely used to treat riboflavin transporter deficiencies and significantly improves or stabilizes the phenotype of patients, especially if started as soon as symptoms arise [128], but the molecular basis of riboflavin responsiveness is still unknown. A possible explanation could be that the expression of RFVTs is overlapping in most tissues and if one transporter is not functioning properly, the others can take over.

Variants in *SLC52A1*, which encodes RFVT1, highly expressed in the placenta, have been reported in two pregnant women, causing transient MADD (OMIM #615026) in their infants [130,131,132]. Both women had a complicated pregnancy with hyperemesis gravidarum. The first woman carried a heterozygous deletion in *SLC52A1*, which results in a complete disruption of *SLC52A1* expression in the affected allele [130,131]. Biochemical characterization of the woman revealed a mild MADD profile, but no clinical symptoms were detected. At birth, her infant similarly showed a MADD-like biochemical profile and complex neurological symptoms, which were fully normalized shortly after riboflavin treatment (100 mg/d). The clinical and biochemical abnormalities did not reappear after riboflavin treatment discontinuation and genetic analyses did not reveal the *SLC52A1* deletion found in the mother. During the second pregnancy, the woman was supplemented with riboflavin and the second infant did not experience transient MADD.

In 2017, Mosegaard et al., reported a second case [132]. A heterozygous *SLC52A1* intronic variant (c.1134 + 11G>A) was detected both in the mother and in the infant. In vitro studies demonstrated that the variant creates a binding site for the splice inhibitory hnRNP A1 protein, causing exon 4 skipping and most likely nonsense-mediated decay (NMD) of the resulting transcript. At birth, the infant harbored a MADD-like clinical and biochemical phenotype which was corrected with vitamin and carnitine supplementation, including riboflavin (100 mg two times a day), and a low fat and protein diet. The riboflavin treatment was discontinued at 2.5 years of age with no reappearance of MADD metabolites or symptoms, confirming the transient phenotype. The mother showed neither MADD biochemistry nor clinical symptoms, but borderline low FAD/riboflavin levels were detected in her blood shortly after parturition. These two case reports show that low placental transport of riboflavin due to RFVT1 haploinsufficiency, can cause transient MADD in newborns that can be easily corrected, and prevented, by riboflavin supplementation. Catabolic stress, induced by hyperemesis gravidarum, seems to be an important risk factor.

#### 4.4.2. Mitochondrial Folate Transporter Deficiency

Another disorder of flavin transport that can be easily corrected by riboflavin supplementation is riboflavin-responsive exercise intolerance (RREI; OMIM #616839), caused by inborn genetic errors in *SLC25A32*, encoding MFT. Two patients harboring *SLC25A32* variants have been reported up to now. The first patient was reported in 2016 by Schiff et al. [133]. The 14-year-old girl experienced recurrent exercise intolerance and biochemical analyses showed a MADD-like profile, reflecting ETF/ETF-QO and/or ACADs dysfunction. Skeletal-muscle biopsy revealed ragged-red fibers and lipid storage together with a faint succinate dehydrogenase staining. Genetic analyses revealed two heterozygous variants in *SLC25A32*, one missense variant and one nonsense variant. A second patient was described in 2017 by Hellebrekers et al. The 52-year-old patient experienced ataxia, myoclonia, dysarthria, muscle weakness and exercise intolerance [134]. Similar to Schiff et al., the patient showed a MADD-like biochemical profile and impaired ETC function. Genetic analyses revealed a homozygous deletion-insertion in *SLC25A32*, which comprehended the start codon. Both patients were treated with riboflavin, which improved their phenotype. Riboflavin supplementation most probably restores mitochondrial FAD levels, but the mechanisms are unclear, especially for the patient harboring a homozygous LOF variant, which is not easily explained unless disrupted mitochondrial FAD import is compensated by mitochondrial FAD synthesis or other mechanisms ensuring mitochondrial FAD supply.

#### 4.4.3. FAD Synthase Deficiency

Riboflavin supplementation is currently used as a treatment also in lipid storage myopathy due to flavin adenine dinucleotide synthetase deficiency (LSMFLAD; OMIM #255100), caused by pathogenic variants in *FLAD1*, the gene encoding FADS [9]. FADS is a bi-functional enzyme with a N-terminal molybdopterin-binding domain (MPTb) and a C-terminal 3′phosphoadenosine 5′phosphosulfate reductase domain (PAPS). The PAPS domain is able, per se, to catalyze FAD synthesis [135]. Two isoforms, a mitochondrial (FADS1) and a cytosolic (FADS2) one, have been described in detail, and a shorter isoform (FADS6), which contains the sole PAPS domain, has been recently characterized at the protein level and shown to have FAD synthase activity [6,136]. In 2016, *FLAD1* variants were outlined in nine individuals with a MADD-like biochemical profile [9,137]. Six novel patients have been reported in the last four years and new patients are under investigation [138,139,140,141,142]. The phenotype of patients is extremely heterogeneous, ranging from early-onset and severe cardiac and respiratory insufficiency, often with a poor prognosis, to late-onset myopathies. Interestingly, patients with biallelic LOF variants in the MPTb domain (exon 2) harbored residual FADS activity and a considerably high cellular FAD level [9,139]. The expression of FADS6, encoded by a downstream ATG in exon 2, could be a possible explanation [9,136]. ETF, ETF-QO, ACADs and ETC decompensation have been observed in the patients at various extent, as a direct consequence of FAD depletion [9,138,139,142]. Interestingly, in LSMFLAD patients both FMN (FADS substrate) and FAD (FADS product) are decreased [9,139]. This suggests that FADS dysfunction could exert a negative feedback control on either RFK or RFVT2, but the molecular mechanisms are unknown [9,139]. FAD, FMN and riboflavin depletion has been observed in *C. elegans* with silenced *flad1* gene (*FLAD1* orthologue), further supporting this hypothesis [143].

Riboflavin supplementation has shown to be beneficial in patients harboring mild missense variants in the PAPS domain and a mild myopathic phenotype [9,137,144], while it has no or limited effect in patients with biallelic LOF variants and a severe phenotype [139,140,141,144]. The molecular rationale of riboflavin responsiveness is unclear. In patients with mild missense variants, riboflavin supplementation could rescue folding stability of mutant FADS and/or counteract the negative feedback control on RFK/RFVT2 [9,139]. Boosting FADS6 activity in patients with LOF variants in the MPTb domain could be a possible therapeutic approach in the future [136].

**Table 1:** Overview of riboflavin-responsive disorders.

Disorder	OMIM#	Gene(s) (Gene ID)	Clinical Response	Biochemical Response	References
Multiple Acyl-CoA Dehydrogenase Deficiency (MADD)	#231680	*ETFA* (2108) *ETFB* (2109) *ETFDH* (2110)	Several studies documenting complete or partial recovery in patients with mild missense variants. Not effective in patients with more severe missense variants or biallelic LOF variants	Significant reduction or normalization of multiple urinary organic acids and blood acylcarnitine excretion. Significant increases in ETF-QO protein or fatty acid oxidation flux in patients’ cultured fibroblasts upon supplementation with riboflavin	[75,82]
Short-Chain Acyl-CoA Dehydrogenase Deficiency (SCADD)	#201470	*ACADS* (35)	Two single studies; indications of clinical improvement in 4/16 patients. The four patients were compound heterozygous for c.625G>A susceptibility variant and a rare missense variant. A clear clinical improvement reported in one patient with homozygous c.625G>A susceptibility variant	All patients clinically responding to riboflavin treatment had decreased or normalized urinary organic acids excretion and/or decreased butyrylcarnitine in blood. One patient responded biochemically but not clinically	[83,87]
Medium-Chain Acyl-CoA Dehydrogenase Deficiency (MCADD)	#201450	*ACADM* (34)	NR	Two studies; significant increase of MCAD enzyme activity in cultured lymphocytes from 5/5 patients. The genetic characterization of the patients was not reported. In a study of 17 patients with *ACADM* missense variants, patients supplemented with riboflavin and carnitine (6) did not display blood markers of oxidative damage when compared with carnitine supplemented (7) and non-supplemented (14) patients	[88,89]
ACAD9 Deficiency or Mitochondrial Complex I Deficiency Nuclear type 20 (MC1DN20)	#611126	*ACAD9* (28976)	Several studies. In one comprehensive study, riboflavin treatment resulted in clinical improvement in 20/31 patients. Responsive patients carried missense variants	Significant increase of complex I activity was seen upon supplementation with riboflavin in fibroblasts derived from 9/15 patients	[91]
Mitochondrial Complex II Deficiency	#252011	*SDHAF1* (612848)	Riboflavin treatment resulted in a clinical improvement in 3 patients	Plasma lactate, pyruvate and alanine remained within the control range in two patients. In a third patient, plasma lactate was elevated and normalized after riboflavin treatment	[92,94,95]
Ethylmalonic Encephalopathy (EE)	#602473	*ETHE1* (23474)	Riboflavin treatment, in combination with other vitamins and CoQ10, has in a few cases been seen to slightly reduce symptoms. A single study has reported a partial effect of riboflavin alone	Clear biochemical improvement of blood acylcarnitines has been reported using riboflavin in combination with other vitamins, CoQ10, and NAC. A single study has showed some improvements upon treatment with riboflavin alone	[109,111,112,121]
Brown-Vialetto-Van Laere Syndrome 1 and 2 (BVVLS1; BVVLS2), Fazio-Londe Disease	#211530 #614707 #615026	*SLC52A3* (113278) *SLC52A2* (79581)	Several studies; clinical improvement or stabilization of symptoms observed in almost all patients treated with riboflavin	Some patients have shown abnormalities in blood acylcarnitines, urine organic acids, and plasma flavin content that were improved by riboflavin treatment	[128,129,145]
Transient MADD or Riboflavin Deficiency	#615026	*SLC52A1* (607883)	Two cases; clear clinical improvement of both neonates	Biochemical normalization of blood acyl-carnitines and/or urine organic acids in both mothers and neonates	[130,131,132]
Mitochondrial Folate Transporter Deficiency or Riboflavin-Responsive Exercise Intolerance (RREI)	#616839	*SLC25A32* (81034)	Clinical improvement in 2/2 patients	NR	[133,134]
Lipid Storage Myopathy due to Flavin Adenine Dinucleotide Synthetase Deficiency (LSMFLAD)	#255100	*FLAD1* (80308)	Several cases; clinical improvement in patients harboring missense variants. Patients with biallelic LOF variants have shown only a transient clinical improvement or an alleviation of symptoms	Some decreases in acylcarnitine species and normalization of urine organic acids in patients harboring missense variants	[9,88,137,139,141,144]

Abbreviations: CoQ10, Coenzyme Q10; LOF, loss of function; NAC, N-acetylcysteine; NR, not reported.

## 5. Conclusions and Perspectives for Research and Clinical Managements of IEM

Riboflavin deficiency has implications for human health due to the key role of its derivatives, FMN and FAD, in various essential cellular functions and physiological conditions, as described above. Whilst clinical riboflavin deficiency is rare in developed countries, riboflavin insufficiency and subclinical riboflavin deficiency is widespread and could give rise to clinical manifestations when combined with an increased need of riboflavin due to physiological conditions like pregnancy, exercise, infections, or due to genetic disorders of flavin biosynthesis and function.

Since the first riboflavin-responsive metabolic defect was described in 1982 as a mild variant form of MADD [146], an increasing number of riboflavin-responsive IEM have been identified and characterized. The response to riboflavin has been explained by the ability of riboflavin to compensate for genetic deficiencies in riboflavin transport or cofactor synthesis [9,131,133,147,148], or by the ability of riboflavin-derived cofactors to improve folding and stability of individually mutated flavoproteins [9,65,69,149]. In addition, *ETFDH* and *FLAD1* variants could induce secondary disturbances of cellular flavin homeostasis, potentially creating a vicious cycle of impaired mitochondrial electron transfer [9,69,77,143], whose exact mechanism is still under investigation. 

Riboflavin deficiency induced by pregnancy and/or low vitamin supply can reveal milder genotypes or carriers, as described for transient MADD [130,132], where both women displayed hyperemesis gravidarum and an increased risk of low nutrient and vitamin supply. Moreover, one of the reported *SLC52A1* variants (c.1134 + 11G>A) is a mild splice variant with a rather high allele frequency in the general population (MAF 0.2%). This variant could represent a risk factor for transient MADD in newborns when combined with insufficient riboflavin supply during pregnancy. Similar, heterozygous *ETFDH* variants were recently reported in two elderly patients with stress-induced myopathy and lack of a previous history of myopathy. The authors speculate that non-genetic factors, such as statin therapy and hypothyroidism, could have acted as precipitating factors and thereby triggered the clinical symptoms [150]. Nevertheless, the clinical symptoms and biochemical defects fully reversed after treatment with riboflavin and carnitine. This, combined with a decreased efficiency of riboflavin absorbance or intake in the elderly population [3,34,35,36,37], could further support the role of disturbed riboflavin supply in the pathogenesis of the disease. Additional cases of clinically manifesting *ETFDH* carriers, highly responsive to riboflavin therapy, are known to the authors of this paper and may represent an under-recognized phenomenon. 

The survival rate of patients with IEM has remarkably improved in the last 40 years due to better diagnosis and treatment options, including the implementation of expanded newborn screening programs. However, elderly people and the majority of women that are currently in their childbearing age, have been born prior to the expanded newborn screening programs. It is also not known to what extent diagnostic metabolites in newborns with riboflavin-responsive genetic variants are initially blurred or masked by transfer of maternally derived riboflavin. The clear benefits, and in some cases lifesaving effects, of high dose riboflavin therapy makes these conditions important to be aware of and to recognize and treat accordingly. A wider use of metabolic and extended genetic screening in suspected cases, together with improved knowledge on the complex gene-environmental interactions that control cellular riboflavin homeostasis, as discussed in the present review, will hopefully add to this. 

## Abbreviations

ACAD	Acyl-CoA Dehydrogenase
AIF	Apoptosis Inducing Factor
AMID	Mitochondrion-Associated Inducer of Death
CHDH	Choline Dehydrogenase
CoQ10	Coenzyme Q10
CytC	Cytochrome C
DMGDH	Dimethylglycine Dehydrogenase
ETC	Electron Transport Chain
ETHE1	Ethylmalonic Encephalopathy Protein 1
ETF	Electron Transfer Flavoprotein
ETF-QO	Electron Transfer Flavoprotein-ubiquinone Oxidoreductase
FAD	Flavin Adenine Dinucleotide
FMN	Flavin Mononucleotide
GCDH	Glutaryl-CoA Dehydrogenase
GPDM	Mitochondrial Glycerol-3-phosphate Dehydrogenase
HSP	Heat Shock Protein
IBD	Isobutyryl-CoA Dehydrogenase
IEM	Inborn Errors of Metabolism
IL	Interleukin
IVD	Isovaleryl-CoA Dehydrogenase
LCAD	Long-Chain Acyl-CoA Dehydrogenase
LOF	Loss of Function
LSD1	Lysine-specific Demethylase 1
MADD	Multiple Acyl-CoA Dehydrogenation Deficiency
MCAD	Medium-Chain Acyl-CoA Dehydrogenase
MFT	Mitochondrial Folate Transporter
MFTHR	Methylenetetrahydrofolate Reductase
MPTb	Molybdopterin-binding Domain
MTRR	Methionine Synthase Reductase
NADPH	Nicotinamide Adenine Dinucleotide Phosphate
NMD	Nonsense-mediated Decay
NOX	NADPH oxidases
PNPO	Pyridoxine 5´-phosphate Oxidase
RFK	Riboflavin Kinase
RFVT	Riboflavin Transporter
ROS	Reactive Oxygen Species
SARDH	Sarcosine Dehydrogenase
SBCAD	Short/Branched-Chain Acyl-CoA Dehydrogenase
SCAD	Short-Chain Acyl-CoA Dehydrogenase
SLC	Solute Carrier
SQOR	Sulfide:Quinone Oxidoreductase
TNF-α	Tumor Necrosis Factor Alpha
VLCAD	Very Long-Chain Acyl-CoA Dehydrogenase
